# Perceptions of Psychologists in Spanish Public Health Centers Regarding the Well-Being of Patients With Anxiety Complaints

**DOI:** 10.7759/cureus.100188

**Published:** 2025-12-27

**Authors:** Geane Uliana Miranda, Valeschka Martins Guerra, Maria Cristina Menandro, Rosa Baños

**Affiliations:** 1 Department of Social and Developmental Psychology, Federal University of Espírito Santo, Vitória, BRA; 2 Department of Personality, Assessment and Psychological Treatments, University of Valencia, Valencia, ESP

**Keywords:** anxiety, mental health, patients, psychologists, well-being

## Abstract

Persistent anxiety impairs quality of life and calls for approaches beyond symptom reduction. The Positive Mental Health framework expands this perspective by emphasizing subjective, psychological, and social dimensions of well-being. This research aimed to examine how psychologists working in Spanish public health services perceive the well-being of patients with anxiety, focusing specifically on clinicians’ interpretations rather than patients’ self-reports. This qualitative, exploratory study used semi-structured interviews to investigate these perceptions, gathering accounts from psychologists employed in public health centers across Spain. Twelve professionals, mostly from the Valencian Community, participated in semi-structured interviews conducted predominantly in an asynchronous online format. Data were analyzed using thematic-categorical content analysis and subsequently organized into two analytical axes comprising eleven categories. The first axis, general perspectives, encompassed: characteristics of patients and society; risk factors; types and symptoms of anxiety; and professional practice. The second axis, well-being, comprised the categories Presence of Subjective Well-Being (SWB), Absence of SWB, Presence of Social Well-Being (SoWB), Absence of SoWB, Presence of Psychological Well-Being (PWB), and Absence of PWB. While difficulties across well-being domains were prevalent, some accounts showed signs of flourishing. Beyond symptom-focused care, participants described strategies to foster positive functioning. The findings highlight the need for clinical practices that combine empathic listening with active promotion of well-being, acknowledging social influences. Flourishing may emerge even in distress when supported by connection, purpose, and public commitment to mental health.

## Introduction

According to the Diagnostic and Statistical Manual of Mental Disorders, Fifth Edition (DSM-5) [1] and the International Classification of Diseases, 11th Revision [2], anxiety is defined as an affective state characterized by the anticipation of a future or diffuse threat. It commonly involves apprehension, persistent worry, and physiological manifestations such as muscle tension, restlessness, hypervigilance, and a tendency toward caution or avoidance. Anxiety is conceptually distinct from fear, which denotes an immediate alarm response accompanied by pronounced autonomic arousal. As a normative human experience, anxiety can serve adaptive functions by signaling the need for preparation and caution; when it is proportional to context, transient, and non-impairing, it does not constitute a disorder [1,2].

In contrast, anxiety disorders are defined by excessive or persistent fear and worry, heightened physiological arousal, and clinically significant functional impairment. Their etiology is multifactorial, involving biological mechanisms (e.g., GABAergic and serotonergic dysregulation, limbic system alterations, genetic vulnerability), environmental influences (stressors, learned responses, hyperreactivity to threat), and psychosocial factors (family context, chronic stress, maladaptive cognitions). Comorbidity with mood disorders is common [3].

Anxiety constitutes an adaptive response to perceived threats; however, when persistent and intense, it may evolve into a dysfunctional condition that significantly impairs quality of life [4]. In such cases, public health strategies must extend beyond symptomatic relief to encompass interventions that actively promote psychological well-being (PWB). This shift calls for a redefinition of psychological care, one that transcends symptom remission and instead cultivates human flourishing.

Although anxiety has been explored through diverse theoretical lenses, there remains a notable gap in studies addressing its intersection with Positive Mental Health, a framework grounded in Positive Psychology and dedicated to the scientific investigation of human potential [5]. This model, developed by Corey Keyes, reconceptualizes mental health not merely as the absence of psychopathology, but as the presence of positive indicators of functioning organized into three dimensions: subjective well-being (SWB), PWB, and social well-being (SoWB) [6,7]. SWB refers to the hedonic dimension of well-being, encompassing life satisfaction and the experience of positive emotions [8]. PWB, based on Ryff’s eudaimonic framework, includes self-acceptance, autonomy, personal growth, environmental mastery, purpose in life, and positive relationships, reflecting optimal individual functioning [9]. SoWB, as proposed by Keyes within a eudaimonic perspective, comprises social acceptance, social actualization, social coherence, social contribution, and social integration; the combination of high levels across one hedonic and six eudaimonic dimensions defines a state of flourishing, whereas low or absent levels denote stagnation [6].

Keyes’s research has shown that flourishing serves as a protective factor against psychological and physical disorders, while enhancing resilience, engagement, and overall functioning [6,10,11]. These findings underscore the importance of clinical and public health strategies that integrate both prevention and the proactive cultivation of well-being [12].

Nonetheless, investigations into how psychologists perceive the well-being of patients with anxiety remain scarce, particularly those adopting a perspective that extends beyond a strictly pathological lens. This gap is especially consequential in public health settings, where translating the principles of Positive Mental Health into everyday clinical routines continues to pose significant challenges. Within this landscape, Spain offers a particularly informative context: its consolidated public healthcare system and the recent incorporation of psychologists into Primary Health Care (PHC) - 419 professionals as of 2022 - create conditions uniquely suited for examining how well-being constructs are understood and mobilized in practice [13].

Accordingly, this study aimed to explore how psychologists working in Spanish public health services perceive the subjective, psychological, and SoWB of patients with anxiety, within the Positive Mental Health framework. This is an exploratory investigation focused specifically on clinicians’ perceptions, rather than patients’ self-reported experiences, with the goal of generating insights to inform more attuned and well-being-oriented psychological practices in public healthcare settings.

## Materials and methods

Study design

This research employed a qualitative and exploratory design, selected for its suitability in capturing meanings, experiences, and subjective interpretations in clinical contexts [14], and was coordinated at the University of Valencia with the participation of psychologists working in various public health services in Spain, especially in Valencia.

Participants

Inclusion criteria specified that only psychologists employed in the public health system, actively engaged in clinical duties, and willing to participate voluntarily were eligible for the study. Exclusion criteria encompassed psychologists working exclusively in the private sector, those on leave or otherwise inactive during the data collection period, and individuals who declined participation.

No statistical sample size calculation was performed, as this is a qualitative, exploratory study. A purposive sampling strategy was adopted. The final sample of 12 participants was defined based on informational adequacy: after the 10th interview, no substantially new thematic elements emerged, and two additional interviews were conducted to confirm data saturation.

Data collection instruments and procedures

Data collection consisted of semi-structured interviews, most of which were administered asynchronously online, while a smaller number were conducted on-site at the University of Valencia. All interviews followed a script comprising 16 open-ended questions targeting the dimensions of SWB, PWB, and SoWB [6]. The interview guide used in this study was adapted from an instrument originally developed by the authors for a related qualitative investigation with psychologists working in Brazilian public health services. As that study is still unpublished, it cannot be formally referenced; however, the instrument was theoretically grounded in Keyes’ Positive Mental Health framework and in the broader literature on subjective, psychological, and SoWB in the context of anxiety care. The Brazilian version was pilot-tested with psychologists through focus groups to assess clarity, relevance, and applicability to clinical practice, and the resulting feedback informed refinements to the wording and structure of the guide.

For the present study, the interview guide was translated into European Spanish. The translation was carried out by the authors and subsequently reviewed by four Spanish clinical psychology researchers from the University of Valencia. This expert review focused on ensuring semantic clarity, correcting linguistic inaccuracies, and verifying that the wording was appropriate for the clinical and organizational reality of the Spanish National Health System. 

A characterization questionnaire was also developed to collect contextual information on participants (e.g., age, workplace setting, employment status, therapeutic orientation, years of professional experience, and experience treating anxiety). Its content was derived from standard variables commonly used in mental health services research and was reviewed by the same expert panel for face and content validity. Given the qualitative and exploratory nature of the study, no formal psychometric validation procedures were undertaken for either instrument.

Most interviews were administered electronically via Google Meet (Google, Inc., Mountain View, CA) and took place between July 5, 2024, and October 27, 2024, with an average duration of 19 minutes. A smaller subset of interviews was conducted in person at the University of Valencia to accommodate participants who preferred on-site participation. In addition, participants completed a characterization questionnaire administered via Google Forms, which collected sociodemographic (e.g., age, city of residence) and professional information (e.g., type of employment, workplace setting, therapeutic orientation, postgraduate training, experience with anxiety cases). The recruitment strategy combined outreach through social media, professional networks, and a snowball sampling technique, whereby initial participants referred others to the study.

Data processing and analysis

All interviews were fully transcribed and analyzed using thematic-categorical content analysis [15], drawing on additional methodological contributions from Mendes and Miskulin [16] and Oliveira [17]. The analysis was conducted manually rather than with qualitative data analysis software. This decision is consistent with the exploratory nature of the study and the size of the dataset, and allowed for a closer, iterative engagement with the material, particularly relevant in interpretive qualitative designs, where meaning construction depends on sustained, reflexive interaction with the data.

The coding and categorization were performed by one researcher, a clinical psychologist with training in qualitative methods. Although multiple coders are not a formal requirement in thematic-categorical approaches, especially in interpretive paradigms, analytic rigor was strengthened through systematic memo-writing, constant comparison, and multiple cycles of re-reading to ensure internal coherence and traceability of decisions. This strategy aligns with established recommendations in qualitative research for ensuring trustworthiness when a single analyst conducts the interpretation.

In the pre-analysis phase, the research corpus, comprising transcripts of the 12 interviews, was subject to an initial reading to gain a broad understanding of the data and identify emerging insights regarding how psychologists perceive well-being in patients with anxiety.

The exploration phase involved identifying and segmenting recording units (relevant text excerpts) and context units (surrounding elements providing interpretive context). This process enabled the detection of recurring themes associated with patient profiles, symptom expression, clinical practices, and references - explicit or implied - to the three well-being dimensions defined by Keyes [6].

During the interpretation phase, the coded content was organized into thematic axes and analytical categories using systematic grouping criteria: mutual exclusivity, exhaustiveness, theoretical relevance, and internal consistency [15]. This analytical structure resulted in two major thematic axes - general perspectives and well-being - encompassing a total of nine categories, thereby supporting a robust and coherent interpretation aligned with the study’s objectives.

All participant statements were grammatically corrected during transcription solely to enhance clarity and fluency, without altering their original meaning.

Ethical considerations

This study received approval from the Human Research Ethics Committee of the Experimental Research Ethics Board at the University of Valencia, Spain (approval code: 2024-PSILOG-3254839). All participants provided informed consent, in accordance with the ethical principles outlined in the Declaration of Helsinki [18].

## Results

Before presenting the thematic findings, Table 1 summarizes the sociodemographic and professional characteristics of the 12 psychologists who participated in the study. The table provides an overview of age distribution, employment status, work setting, professional experience, therapeutic orientation, and postgraduate training. A brief narrative description follows to contextualize these characteristics within the sample.

**Table 1:** Sociodemographic and professional characteristics of participants (N = 12)

Variable	Category/Values	n (%)/M (SD)
Age (years)	Range	24-59
	Mean (SD)	38.67 (13.08)
Region of residence/work	Valencian community	11 (91.67%)
	Other regions	1 (8.33%)
Employment status	Fixed-term contract	7 (58.33%)
	Public servant/statutory	3 (25%)
	Residency	1 (8.33%)
	Permanent contract	1 (8.33%)
Work setting	Primary Care - Health Center	1 (8.33%)
	Secondary Care - Mental Health Units	3 (25%)
	Hospital-based	5 (41.67%)
	Of which oncology hospitals	4 (33.33% of total sample; 80% of hospital workers)
	Rotating across Primary, Secondary, and Tertiary Care	3 (25%)
Experience in current services	Range	5 months-32 years
	Mean in months (SD)	131.92 (134.04)
Other professional roles	None	8 (66.67%)
	Teaching/research involvement	3 (25%)
Experience with anxiety care	Range	1-32 years
	Mean in months (SD)	154.5 (131.7)
Therapeutic orientation	Cognitive behavioral therapy	10 (83.33%)
	Integrative/eclectic approaches	11 (91.67%)
Postgraduate training	Holds or is completing postgraduate studies	12 (100%)
	Reports that training supported anxiety care	11 (91.67%)

Twelve psychologists participated in the study, ranging from 24 to 59 years of age (M = 38.67; SD = 13.08). Most resided and worked in the Valencian Community (91.67%) and were employed under fixed-term contracts, public service appointments, residency positions, or permanent contracts. Participants worked across different levels of care within the Spanish public health system: one in Primary Care (Centro de Salud), three in Secondary Care (Mental Health Units), five in Tertiary Care (hospital-based services) - four of whom worked specifically in oncology hospitals - and three in rotating positions spanning Primary, Secondary, and Tertiary Care. Professional experience within these services ranged from five months to 32 years. Most participants did not engage in concurrent private practice, although some were involved in teaching or research. Cognitive Behavioral Therapy was the predominant therapeutic orientation, yet nearly all clinicians reported integrating strategies from multiple theoretical frameworks. All participants held or were completing postgraduate training, and most indicated that such training enhanced their clinical management of anxiety.

The presentation of the results adheres to the structure of the thematic axes and corresponding analytical categories, as established during the content analysis and summarized in Table 2. 

**Table 2:** Thematic axes, analytical categories, descriptions, and illustrative quotes from psychologists working in Spanish public health centers PWB = psychological well-being; SoWB = social well-being; SWB = subjective well-being

Axis	Category	Description	Example
General perspectives	Characteristics of patients and society	Sociodemographic profile of patients and contextual societal factors influencing anxiety.	"I think society, with this whole idea of always being okay and that people don't have problems, and that, well, these images on social media and all that aren't flattering. Well, they actually encourage mental health issues, specifically anxiety, and not being what society pushes me to be."(P1).
	Risk factors, types and symptoms of anxiety	Presence of comorbidities, suicidal behavior, cancer diagnoses, economic adversity, typologies of anxiety, and symptomatic manifestations.	“Well, in general, what I see is a lot of comorbidity with depressive symptoms, both anxiety and depression going hand in hand.”(P8).
	Professional practice	Clinical interventions, therapeutic goals, and strategies employed by psychologists in response to anxiety.	“And in fact, I personally find it very motivating to bring that (detection in personal growth) to consult to find reasons to get involved in stagnation, right?”(P8).
Well-being	Presence of SWB (hedonic)	Manifestations of vitality, life satisfaction, and the experience of positive emotions.	“So I think that in general it is usually seen that are, in general, very interested in life” (P8).
	Absence of SWB (hedonic)	Evidence of dissatisfaction with life, reduced vitality, and prevalence of negative affect.	“Most people feel dissatisfied, regardless of what they have and how they live. Precisely because of these ways of judging, then, most feel dissatisfied."(P6).
	Presence of SoWB (positive social functioning - eudaimonic)	Perception of social acceptance, actualization, coherence, contribution, and integration.	"Of course, the more social support they perceive and the more they feel part of a group, we do see a direct relationship, and their anxiety decreases significantly because hypervigilance is diminished. And ultimately, they tend to feel more comfortable with themselves as well."(P7).
	Absence of SoWB (positive social functioning - eudaimonic)	Difficulty in achieving or perceiving social acceptance, actualization, coherence, contribution, and integration.	"Well, I think they complain, but they don't consider themselves part of society, so... They don't consider themselves part of it, they don't get involved, they can't improve, they just complain, right? Instead of asking themselves, 'What can I do for the community? What can I do to improve instead of complaining?' And I think they think more about their own problems, about their individuality."(P10).
	Presence of PWB (individual positive functioning - eudaimonic)	Presence of self-acceptance, autonomy, personal growth, environmental mastery, positive relationships or meaning in life.	“There are some who do have satisfying and trusting relationships.”(P6).
	Absence of PWB (individual positive functioning - eudaimonic)	Difficulty in achieving self-acceptance, autonomy, personal growth, environmental mastery, satisfying relationships, or life purpose.	“I would say that generally no, they don’t have satisfactory relationships.”(P5).

In Axis I - General Perspectives, participants provided insights into the profile of patients and broader societal dynamics, the risk factors associated with anxiety, the most prevalent anxiety symptoms, and the clinical practices adopted by psychologists. These narratives were organized into three analytical categories: (1) characteristics of patients and society, (2) risk factors, types, and symptoms of anxiety, and (3) professional practice.

Characteristics of patients and society

To begin with, this category encompasses sociodemographic data such as age, gender, educational background, and socioeconomic status. As one participant noted, “Most patients are still between 30 and 55” (P3); another added, “I would tell you that it is true that there is still a greater predominance of women” (P2). Additionally, one professional observed: “People who often don't have higher education, who have lower socioeconomic status. I suppose this is also related to why they seek care through the National Health System rather than the private sector” (P6).

Participants also reflected on the broader relationship between contemporary society and mental health, particularly anxiety. As one psychologist put it: “I think the way our society works today increases anxiety in patients with anxiety (...) how catastrophic everything is. The economic problems, problems with wars, problems with unemployment, housing” (P11).

Risk factors, types, and symptoms of anxiety

Another relevant point concerned the risk factors associated with anxiety, including the presence of comorbidities such as depression, suicidal ideation, and cancer. As one participant stated: “People who may have an anxiety disorder or a mood disorder, such as depression or adjustment disorder, who also have anxiety as a comorbidity” (P12). Others highlighted how anxiety can escalate in the presence of severe distress: “But I think that if there is severe and limiting anxiety, there comes a time when thoughts of death appear” (P11); “A person who is anxious will react with much more anxiety to making choices in a situation they cannot control, like cancer” (P9). Additionally, adverse financial conditions were frequently mentioned as triggers or intensifiers of anxiety: “If you are in complex socioeconomic conditions, how can you not be anxious, right?” (P12).

Participants shared insights into various types of anxiety disorders, including social anxiety disorder, specific phobias, and agoraphobia. One psychologist explained: “Or with a more social anxiety, like social phobia, they tend to be more insecure people” (P12). Another noted: “It could be a specific phobia or something like that, but no, I don't see them having any more problems when it comes to expressing their point of view” (P12). In relation to agoraphobia, a participant remarked: “If I have agoraphobia and need others to do things for me, then obviously I probably also had difficulties with the people around me” (P2).

Regarding anxiety symptoms, participants described those most commonly observed in patients, encompassing physical, cognitive, and emotional manifestations: “Physical complaints, which are related to bowel or eating problems, insomnia (...) complaints of ruminative anxiety, of generalized constant worry” (P6).

Professional practice

Finally, participants reflected on their professional practice, addressing both therapeutic goals and clinical direction. In terms of objectives, psychologists emphasized the importance of equipping patients to manage, rather than eliminate, anxiety: “The ultimate goal, I think we share this with patients, is not to reduce it (anxiety), but to learn to manage it” (P6). As for therapeutic direction, they highlighted the value of providing tools for emotional self-regulation: “And in fact, many times, as I said before, we try to give them tools or ways to cope with their anxiety, to manage it, to learn to handle it better and self-regulate” (P12); “Perhaps the most sensible thing to do is to start by seeing what they want to achieve in their lives and from there begin the therapeutic approach” (P11).

Psychologists described clinical practices that support the development of positive functioning, particularly at the individual level. Although they did not explicitly refer to the concept, their accounts align with efforts to promote dimensions of PWB. For instance, they reported interventions aimed at fostering self-acceptance: “I try to work with them to restructure that image (...) improve that image of themselves” (P6); autonomy: “Working on all those aspects of fear and assertiveness, improving communication skills, and to do so, working on the fear of being assertive, the consequences you fear when expressing your opinion assertively” (P2); and personal growth: “To be able to continue guiding you toward your development as a person, the goals that are valuable to you, the role you want to play in society, and the actions that are valid for you” (P12). The dimension of life purpose also emerged as a therapeutic focus: “Often this is part of the treatment, as is working with values and a sense of purpose” (P3). Furthermore, some professionals reported initiatives that support positive social functioning, notably in the area of social contribution: “What if we shifted the focus outward, toward the society you're part of, on what you'd like to contribute to society? That's also sometimes considered in treatment” (P2).

Axis II - Well-Being presents data grounded in the theoretical framework, specifically regarding hedonic indicators (SWB) and dimensions of positive functioning (PWB and SoWB). The material related to these types of well-being was organized into six analytical categories: (1) presence of SWB, (2) absence of SWB (hedonic well-being), (3) presence of SoWB, (4) absence of SoWB (social/eudaimonic functioning), (5) presence of PWB, and (6) absence of PWB (individual/eudaimonic functioning).

Presence of SWB (hedonic)

Participants described the presence of SWB in patients, particularly through indicators such as life satisfaction - “Afterwards, they were able to reevaluate the same situations and feel satisfied” (P7) - and positive affect, albeit in a sporadic form - “You have a moment of happiness. I don't know, your son comes and gives you good news. Or I have patients who say that my granddaughter was born, and they're happy. Great. And you have a moment of happiness” (P2). From the data, it is possible to infer that mild anxiety may coexist with higher levels of positive affect, such as vitality: “I think if we're talking about a mild to moderate anxiety component, I do believe that they are interested in living life, in overcoming their problem, in continuing their life with their life goals” (P11).

Absence of SWB (hedonic)

The absence of SWB emerged more frequently in participants’ accounts, indicating that patients often report life dissatisfaction - “There’s no satisfaction, and in the end, anxiety can be very limiting, right?” (P3) - and a predominance of negative affect - “Interested in life? You can't; anxiety prevents you from seeing what's important” (P10); “No. Happiness, no. (...) Happiness, as understood as life satisfaction, is a bit like what we were talking about before; it depends on the level of anxiety” (P7). In this context, anxiety appears as a barrier to connecting with everyday life and meaningful experiences.

Presence of SoWB (social/eudaimonic)

Participants reported the presence of SoWB in patients, especially in the dimension of social acceptance: “They are empathetic toward others. We see that a lot in therapy groups when we do transdiagnostic groups” (P12). Many patients demonstrate empathy in therapeutic settings, where contact with others experiencing similar forms of suffering fosters emotional bonds. In relation to social integration, psychologists stated that some patients remain connected to their community: “Especially among older women… they usually have acquaintances and others who support them within their circle” (P8). However, no clear examples were identified regarding social actualization, social coherence, or social contribution, suggesting difficulties in believing in societal progress, making sense of social life, or engaging in community-oriented actions.

Absence of SoWB (social/eudaimonic)

The absence of SoWB emerged more prominently in participants' narratives. With regard to social acceptance, psychologists observed that patients often struggle to embrace differences and complexities: “When they are very overwhelmed… they have more problems understanding the other” (P3). As for social actualization, participants reported frequent disbelief in society's potential for positive change: “Is it getting better? No… They just complain” (P10). In terms of social coherence, many patients struggle to make sense of social structures and dynamics: “Some of the things in society don't make sense to them” (P2). Concerning social contribution, participants indicated reduced perceptions of usefulness: “I’m not contributing enough… I have nothing to contribute” (P4). Finally, with respect to social integration, feelings of exclusion were common: “They feel rejected by their community…” (P6).

Presence of PWB (individual/eudaimonic)

Participants reported the presence of PWB. For self-acceptance, some patients showed more compassionate self-views: “If they can encapsulate anxiety as a part of themselves… they’re able to value other strengths” (P11). For personal growth, professionals observed recognition of development: “Managing and learning to live with anxiety… they are proud of how they've done it” (P7). For positive relationships, many maintained meaningful connections: “They have some connection… someone who accompanies them” (P10). For purpose in life, some linked meaning to spirituality: “People with a certain degree of spirituality… go beyond themselves” (P10). Although not universal, these accounts suggest routes for cultivating purpose in clinical work.

Absence of PWB (individual/eudaimonic)

The absence of PWB was more prominent. Regarding self-acceptance, psychologists observed negative self-perceptions and low self-esteem: “Most people report low self-esteem” (P1); “…lower level of effectiveness than people who don't have anxiety problems” (P2). Substantial difficulties also appeared in autonomy: fear of social evaluation often inhibited asserting opinions - “People are very concerned about the social evaluation of others” (P6). For personal growth, reports of stagnation were recurrent: “They often feel stuck” (P1). In environmental mastery, two contrasting profiles emerged - hyper-responsibility versus avoidance: “A very hyper-responsible patient… Another profile… avoiding it” (P8).

Finally, many participants described positive relationships marked by emotional disconnection and invalidation: “People around them find it difficult to understand or support them” (P3); “Interpersonal problems” (P4). As for purpose in life, several patients “survive more than they live,” without clear meaning (P6).

## Discussion

Axis I - general perspectives

The participant’s perceptions are consistent with the findings of Aguilar-Palacio et al. [19], whose study revealed a higher prevalence of poor self-perceived health among women and individuals with lower levels of education.

These reflections resonate with Expósito-Duque et al. [20], who identify structural and social factors as key contributors to the rise in anxiety, and argue that PHC must also play a preventive role, minimizing the overuse of medication.

The literature corroborates the strong association between anxiety disorders and insomnia, recognizing the effectiveness of targeted interventions in managing this symptom [21]. It also highlights rumination and excessive worry as frequent cognitive features [22].

In parallel, empirical evidence shows that psychological interventions - such as Positive Psychology programs and contemplative practices - can effectively enhance PWB dimensions [23], suggesting that anxiety management may benefit from approaches that integrate both symptom reduction and the cultivation of well-being.

In summary, the findings from Axis I underscore the multifaceted nature of anxiety within the context of psychological care in public health. The first category illustrates the interplay between social markers - such as gender, education, and socioeconomic status - and manifestations of psychological distress, underscoring the need for clinically attuned and socially informed listening. The second category highlights the intricate web of risk factors, symptoms, and comorbidities - particularly depression and financial hardship - that intensify anxiety and hinder the experience of well-being. Lastly, participants described professional practices oriented toward fostering positive psychological functioning, demonstrating an integrative approach that connects social determinants, the lived experience of anxiety, and the psychologist’s role in advancing mental health promotion.

Axis II - well-being

The presence of momentary happiness reported by participants aligns with the definition of the positive affect dimension as transient emotional states [24]. The frequent absence of SWB in the accounts is consistent with evidence of a strong association between negative affect, low life satisfaction, and anxiety symptoms [24]. Together, the literature and the data suggest that fostering positive affect and life satisfaction may operate as protective factors against anxiety.

Empathy observed in group settings is coherent with trust and kindness as core components of social acceptance [25]. Community ties noted by participants can enhance belonging and recognition [26]. Conversely, difficulties in social coherence described by participants are compatible with anxiogenic social environments highlighted in recent work on social determinants of anxiety [20]. The reported feelings of exclusion and lack of support reinforce the need for inclusive environments and responsive community systems [25].

Reports of meaningful bonds as buffers against distress converge with evidence linking satisfying interpersonal relationships to lower anxiety levels [27]. The two behavioral poles under environmental mastery (hyper-responsibility vs. avoidance) underscore the importance of interventions that reduce experiential avoidance and promote functional coping [27]. Strengthening self-regard also aligns with literature associating improved self-views with anxiety reduction [28]. Finally, the recurrent absence of purpose in life mirrors findings that greater perceived life purpose relates to lower anxiety, underscoring the value of reconstructing meaning as part of PWB-oriented care [29].

Overall, Axis II indicates that anxiety is deeply entangled with subjective, psychological, and social dimensions of well-being. Occasional signs of flourishing emerged in contexts that foster emotional bonds and inner resources, but the prevailing absence of such indicators points to significant vulnerability, often compounded by adverse social conditions. Flourishing should be understood less as a static state and more as an ongoing process of reconstructing bonds and meaning; promoting well-being thus calls for interventions that integrate subjectivity and sociocultural reality, embracing suffering while cultivating strengths and belonging.

A key strength of this study lies in its use of Keyes’ multidimensional framework to examine clinicians’ perspectives on anxiety, allowing the analysis to move beyond symptom-oriented descriptions toward a more comprehensive account of patients’ functioning. Additionally, interviewing psychologists from multiple levels of the Spanish public health system-primary, secondary, tertiary, and rotating posts-provided a heterogeneous view of clinical realities, enhancing the ecological validity of the findings. The methodological depth afforded by thematic-categorical analysis also enabled a fine-grained examination of how subjective, psychological, and social indicators of well-being manifest in everyday clinical encounters.

These findings generate several practical and clinical implications. First, they suggest that routine anxiety care may benefit from incorporating brief indicators of well-being, such as sense of purpose, perceived social support, and engagement in meaningful activities, alongside traditional symptom monitoring. Second, therapeutic encounters can include short, structured moments to explore what patients value, how they relate to others, and which daily actions help them feel connected or effective. Such conversations, even when brief, may reduce hypervigilance and avoidance by reinforcing experiences of coherence and agency. Third, services at different levels of care could adopt simple and scalable practices - for example, asking patients about one meaningful activity to maintain during the week, identifying one supportive person to contact, or practicing a small self-acceptance exercise-that support well-being without adding substantial workload for clinicians.

These practice-oriented implications should be understood as exploratory and hypothesis-generating, grounded in clinicians’ perspectives within specific Spanish public health settings. They do not constitute prescriptive recommendations, nor do they directly reflect patients’ well-being or treatment outcomes.

As for limitations, the exclusive focus on psychologists’ perspectives highlights the importance of incorporating patient voices in future studies, thus enabling methodological triangulation and a more comprehensive understanding. In addition, although data were collected through semi-structured interviews, the predominantly online modality may have limited opportunities for in-depth probing compared to fully in-person interviews, which should be considered when interpreting the findings. Furthermore, the regional scope, concentrated in the Valencian Community, suggests the potential value of replicating the study in other geographic and healthcare contexts. Accordingly, the findings should be interpreted as contextually grounded and situated within the organizational and cultural features of the settings studied.

## Conclusions

Psychologists in Spanish public health centers describe anxiety as intertwined with patients’ life contexts and report working beyond symptom control to strengthen elements of subjective, psychological, and SoWB. Their accounts suggest that markers of positive functioning can coexist with distress when clinical relationships, continuity of care, and everyday resources are mobilized to sustain connection, purpose, and participation.

These insights point to practical directions: care models that integrate regulation of symptoms with cultivation of strengths; therapeutic settings that foster belonging and meaning; and service routines that protect time for listening and follow-up. Future work should add patients’ perspectives and diversify regions and services to refine how public systems can consistently support movement toward flourishing.

## Appendices

Interview guide

What is the profile of the patients with anxiety-related problems whom you treat? (age range, gender, marital status, socioeconomic level, etc.)

What are the main complaints and anxiety symptoms presented by these patients?

In your opinion, does the way our society functions make sense to patients with anxiety-related problems? a. If yes: “How?/Could you give an example?” If not: “Why not?”

In your opinion, how do patients with anxiety-related problems perceive themselves within their community (their sense of belonging to a social group or neighborhood)?

In your opinion, how do patients with anxiety-related problems perceive their own contribution to society?

In your opinion, do patients with anxiety-related problems believe that society is improving? a. If yes: “How?/Could you give an example?” If not: “Why not?”

In your opinion, do patients with anxiety-related problems hold a positive attitude toward other people, taking into account their differences and complexities? If yes: “How?/Could you give an example?” If not: “Why not?”

In your opinion, do patients with anxiety-related problems have satisfactory and trusting interpersonal relationships? a. If yes: “How?/Could you give an example?” If not: “Why not?”

In your opinion, are patients with anxiety-related problems able to express their point of view with confidence and assurance? a. If yes: “How?/Could you give an example?” If not: “Why not?”

In your opinion, how do patients with anxiety-related problems manage their daily responsibilities?

In your opinion, how do patients with anxiety-related problems perceive their sense of personal growth and development?

In your opinion, how would you describe the way patients with anxiety-related problems accept themselves?

In your opinion, do patients with anxiety-related problems feel interested in life (vitality - hedonic well-being)? a. If yes: “How?/Could you give an example?” If not: “Why not?”

In your opinion, do patients with anxiety-related problems feel satisfied with life, either in general or in specific domains? a. If yes: “How?/Could you give an example?” If not: “Why not?”

In your opinion, do patients with anxiety-related problems have a clear sense of meaning or purpose in life? a. If yes: “How?/Could you give an example?” If not: “Why not?”

In your opinion, do patients with anxiety-related problems feel happy (e.g., joy, good mood, or a sense of calm)? a. If yes: “How?/Could you give an example?” If not: “Why not?”

Participant characterization questionnaire

Do you work in the: Public sector Private sector Both

Age:

City of residence:

City in which you work:

Do you have any postgraduate training (specialization course, master's degree, PhD, etc.)? a. If yes: Do you believe this postgraduate training has helped you better understand how to address anxiety? If so, in what way?

What is your employment status (civil servant, permanent contract, temporary contract)?

In what type of institution do you work (primary health center, specialized unit, hospital, private center, etc.)?

How long have you been working in this institution?

How long have you been working with people with anxiety-related problems?

What is your main therapeutic orientation (e.g., cognitive-behavioral, psychoanalytic, humanistic, etc.)?

Besides your role as a clinical psychologist in the public healthcare system, do you engage in other professional activities (private practice, teaching, research, etc.)?
